# Baicalein, a Bioflavonoid, Prevents Cisplatin-Induced Acute Kidney Injury by Up-Regulating Antioxidant Defenses and Down-Regulating the MAPKs and NF-κB Pathways

**DOI:** 10.1371/journal.pone.0134139

**Published:** 2015-07-29

**Authors:** Bidya Dhar Sahu, Jerald Mahesh Kumar, Ramakrishna Sistla

**Affiliations:** 1 Medicinal Chemistry and Pharmacology Division, CSIR-Indian Institute of Chemical Technology (IICT), Hyderabad, 500 007, India; 2 Animal House Facility, CSIR-Centre for Cellular and Molecular Biology (CCMB), Hyderabad, 500 007, India; National Institutes of Health, UNITED STATES

## Abstract

Acute renal failure is a serious complication of the anticancer drug cisplatin. The potential role of baicalein, a naturally occurring bioflavonoid on cisplatin-induced renal injury is unknown. Here, we assessed the effect of baicalein against a murine model of cisplatin-induced acute renal failure and investigated the underlying possible mechanisms. Renal function, kidney histology, inflammation, oxidative stress, renal mitochondrial function, proteins involved in apoptosis, nuclear translocation of Nrf2 and effects on intracellular signaling pathways such as MAPKs, and NF-κB were assessed. Pretreatment with baicalein ameliorated the cisplatin-induced renal oxidative stress, apoptosis and inflammation and improved kidney injury and function. Baicalein inhibited the cisplatin-induced expression of iNOS, TNF-α, IL-6 and mononuclear cell infiltration and concealed redox-sensitive transcription factor NF-κB activation via reduced DNA-binding activity, IκBα phosphorylation and p65 nuclear translocation in kidneys. Further studies demonstrated baicalein markedly attenuated cisplatin-induced p38 MAPK, ERK1/2 and JNK phosphorylation in kidneys. Baicalein also restored the renal antioxidants and increased the amount of total and nuclear accumulation of Nrf2 and downstream target protein, HO-1 in kidneys. Moreover, baicalein preserved mitochondrial respiratory enzyme activities and inhibited cisplatin-induced apoptosis by suppressing p53 expression, Bax/Bcl-2 imbalance, cytochrome c release and activation of caspase-9, caspase-3 and PARP. Our findings suggest that baicalein ameliorates cisplatin-induced renal damage through up-regulation of antioxidant defense mechanisms and down regulation of the MAPKs and NF-κB signaling pathways.

## Introduction

Cisplatin, a platinum-based inorganic compound, is one of the most potent and most widely used antineoplastic drugs. It is used for the treatment of several human malignancies, including testicular, ovarian, head, neck and lung cancers [1]. Unfortunately, the clinical use of cisplatin as a chemotherapeutic agent is limited by its side effects in normal tissues. Due to preferential accumulation of cisplatin in renal tubules, acute kidney injury is a serious and frequent complication in cancer patients undergoing cisplatin chemotherapy [2]. Despite advances in understanding the pathophysiology of cisplatin-induced toxicities and improved supportive care, in clinical practice, approximately 25–40% patients experience renal dysfunction after treatment with cisplatin [3]. Thus, a great challenge lies ahead in identifying an effective therapy for the prevention of nephrotoxicity associated with cisplatin therapy.

The proposed mechanism of cisplatin-induced renal damage includes the generation of reactive oxygen species (ROS), mitochondrial dysfunction, caspase activation, DNA damage and apoptotic and/or necrotic cell death [1, 4, 5]. Studies from different research group also demonstrated that activation of various stress responsive signaling pathways, such as mitogen-activated protein kinases (MAPKs) in the development and progression of cisplatin-induced renal damage [6]. A number of animal studies have also highlighted a strong contribution of inflammation in the pathogenesis of cisplatin-induced nephrotoxicity [5, 7]. Moreover, ROS are important in enhancing inflammation through the activation of NF-κB and its related signaling pathways [8, 9]. Apart from this, a substantial literature documents the role of Nrf2 (nuclear factor E2-related factor 2) in the regulation of physiological processes that serve to inhibit the development and progression of cisplatin-induced renal damage [10, 11]. It has been reported that absence of Nrf2 exacerbates cisplatin-induced nephrotoxicity and pharmacological activation of Nrf2 has been shown to inhibit cisplatin-mediated nephrotoxicity [10, 12]. Thus, pharmacological activation of Nrf2 is considered as an important molecular target to prevent cisplatin-induced renal damage.

Flavonoids, a group of dietary botanicals with variable phenolic structures, have gained considerable attention in the field of drug discovery and health food supplements [13]. Baicalein is a predominant flavonoid isolated from the roots of *Scutellaria baicalensis* Georgi. Baicalein is a potent antioxidant and displays anti-inflammatory properties in vitro and in vivo [14]. Evidence indicates that baicalein has multiple biological activities, including cardioprotective [15], neuroprotective [16] and hepatoprotective [17]. Previous investigations have shown that baicalein ameliorates lipopolysaccharide-induced glomerulonephritis [18], suppresses radiation-induced inflammatory process in mouse kidneys [19] and attenuates renal dysfunction in type 2 diabetic rats [20]. Baicalein has also been reported to inhibit the proliferation and induce apoptosis in different tumor cells in vitro [21, 22]. Recently, it has also been demonstrated that baicalein increases the cytotoxicity of cisplatin [23] and suppresses tumor cell growth in mouse breast cancer cells [24]. However, the effect of baicalein in kidney protection during cisplatin treatment has never been investigated. Therefore, the present study was undertaken to examine the effects of baicalein on cisplatin-induced renal damage and the underlying mechanisms involved.

## Materials and Methods

### Reagents, chemicals and kits

Cisplatin, baicalein (≥ 98.0% purity), cytochrome c oxidase and superoxide dismutase (SOD) assay kits were purchased from Sigma-Aldrich Co, St Louis, MO, USA. NF-κB (p^65^) transcription factor assay kit was obtained from Cayman Chemical Company, Ann Arbor, MI. NE-PER nuclear and cytoplasmic extraction reagents, Bicinchoninic acid (BCA) protein assay kit and Halt protease inhibitor cocktail were purchased from Pierce Biotechnology, Rockford, IL, USA. Mouse specific TNF-α (BD OptEIA) and IL-6 (BD OptEIA) ELISA kits were obtained from BD Bioscience, San Diego, CA, USA. All other chemicals were of analytical grade.

### Experimental animals and ethics statement

Male Balb/C mice (8 weeks old, weighing 23–25 g) were purchased from National Institute of Nutrition (NIN) (Hyderabad, India) and were maintained under a controlled environment (22±2°C temperature and 55±10% humidity) with 12h light/dark cycle. The animals were acclimatized for 1 week before the study and had free access to standard laboratory feed and water ad libitum. The use of laboratory animals was approved by the Institutional Animal Ethics Committee (IAEC) of CSIR-Indian Institute of Chemical Technology (IICT) and was handled according to the Committee for the Purpose of Control and Supervision of Experiments on Animals (CPCSEA) guidelines, Government of India for safe use and care of experimental animals.

### Experimental design

Animals were randomly divided into 5 groups consisting 8 in each group and were treated as follows: Group I (Control), received 2% gum acacia suspension through per oral route for 15 consecutive days and a single intraperitoneal (i.p) injection of normal saline on 12^th^ day; Group II (Baicalein), received baicalein (50 mg/kg, in 2% gum acacia suspension) through per oral route for 15 consecutive days and a single intraperitoneal (i.p) injection of normal saline on 12^th^ day; Group III (Cisplatin), received 2% gum acacia suspension through per oral route for 15 consecutive days and a single intraperitoneal (i.p) injection of cisplatin (20 mg/kg body weight, dissolved in normal saline) on 12^th^ day; Group IV (Cis+Bai-25), received baicalein (25 mg/kg body weight, in 2% gum acacia suspension) through per oral route for 15 consecutive days and a single intraperitoneal (i.p) injection of cisplatin (20 mg/kg body weight, dissolved in normal saline) on 12^th^ day. Group V (Cis+Bai-50), received baicalein (50 mg/kg body weight, in 2% gum acacia suspension) through per oral route for 15 consecutive days and a single intraperitoneal (i.p) injection of cisplatin (20 mg/kg body weight, dissolved in normal saline) on 12^th^ day. The dose and duration of the baicalein treatment was chosen based on the previously published literature with slight modification, in which baicalein administration at a dose of 150 mg/kg body weight for 14 consecutive days showed a significant protection against lipopolysaccharide-induced glomerulonephritis in mice [18]. Based on this literature, a pilot study was conducted by taking four different doses of baicalein (i.e. 12.5, 25, 50 and 100 mg/kg body weight) to evaluate the effect on cisplatin-induced nephrotoxicity in mice. Baicalein was pre-administered orally through oral gavage for 12 consecutive days prior to cisplatin-induced acute kidney injury and continued for next 3 days (72 h post cisplatin administration) before collection of blood samples to evaluate the serum specific renal function parameters (BUN and creatinine). The results showed that all the selected doses of baicalein (except 12.5 mg/kg) had significant (p< 0.001) ameliorative effect in this cisplatin-induced nephrotoxicity mouse model. Thus, baicalein at a dose of 50 mg/kg and its lower dose, 25 mg/kg were selected for main study to evaluate the dose-dependent effect. The dose of the cisplatin (20 mg/kg body weight) was selected based on the previously used methods [25, 26].

After 72 h of cisplatin administration (i.e. on 15^th^ day), body weight of animals was recorded, blood was collected from the retro-orbital plexus of each experimental animals, the serum was separated by centrifugation (4000 rpm for 15 min) and stored at -80°C until assayed. Then all the animals were euthanized with CO_2_ asphyxiation, kidney tissue samples were dissected and deep frozen in liquid nitrogen to stop metabolic activity, and stored at -80°C for further analysis.

### Assessment of renal function

Serum samples were examined for blood urea nitrogen (BUN) and creatinine in an auto analyzer (Siemens, Dimension Xpand^plus^, USA) using standard diagnostic kits (Siemens, India). In addition, relative weight of kidneys (kidney to body weight ratio normalized to 100 g body weight) was also assessed to evaluate the renal injury.

### Histopathology

Kidney tissue samples were fixed in 10% buffered formalin for 48 h. The samples were de-waxed in xylene and rehydrated in a series of graded alcohols and then embedded in paraffin. These samples were then cut into 5 μm thick sections and stained with hematoxylin and eosin (H and E) for histopathological analysis under light microscope using Zeiss microscope (Axioplan 2 Imaging, Axiovision software).

### Assessment of oxidative stress in the kidney tissues

The levels of reduced glutathione (GSH) [27] and vitamin C [28] and the activities of glutathione S-transferase (GST) [29], glutathione reductase (GR) [30], catalase (CAT) [31], total superoxide dismutase (SOD) (Sigma-Aldrich Co, St Louis, MO, USA) and NAD(P)H: quinone oxidoreductase 1 (NQO1) [32] in kidneys were determined as described in previous literatures. Thiobarbituric acid reactive substance (TBARS), as an index of lipid peroxidation [33] and tissue nitrites levels [34], as an index of tissue nitrative stress were also estimated as described previously. The total protein content in each sample was estimated using Bicinchoninic acid (BCA) protein assay kit (Pierce Biotechnology, Rockford, IL, USA) against bovine serum albumin (BSA) as standard.

### Isolation of renal mitochondrial fraction and the determination of cytochrome c release to cytosol, mitochondrial respiratory and MnSOD enzyme activities

Mitochondrial and cytosolic fractions from the kidney tissue (left side) of all experimental animals were isolated as described in earlier literature [1, 35]. The amount of cytochrome c protein in both cytosol and mitochondria was determined by western blotting to assess cytosolic translocation of cytochrome c. The activity of cytochrome c oxidase (COX) in mitochondrial fraction was determined using cytochrome c oxidase assay kit (Sigma–Aldrich Co., St. Louis, MO, USA) according to the manufacturer’s protocol. Mitochondrial succinate dehydrogenase (SDH) activity was determined spectrophotometrically according to the method that involves oxidation of succinate by an artificial electron acceptor, potassium ferricyanide, as described earlier [36]. In addition, mitochondrial MTT (3-(4, 5-dimethylthiazol-2-yl)-2, 5-diphenyltetrazolium bromide) reduction assay was performed to assess the mitochondrial intactness by incubating the isolated mitochondria with MTT solution (5 mg MTT/ml of 50mM phosphate buffer saline, pH 7.4) for 3h at 37°C [37]. Depending upon the mitochondrial intactness, formazan crystals thus formed were solubilised in dimethyl sulfoxide and optical density was recorded at 580nm. Similarly, the activity of SOD enzyme in the mitochondrial fractions such as MnSOD was measured using SOD activity assay kit (Sigma-Aldrich Co, St Louis, MO, USA) as per manufacturer instructions.

### Determination of TNF-a and IL-6 levels in kidney tissues

The concentrations of TNF-α and IL-6 in kidney tissue homogenates were determined using commercial available kits from BD Bioscience, San Diego, CA, USA as per manufacturer instructions.

### Determination of myeloperoxidase activity in kidney tissues

The activity of myeloperoxidase (MPO) in kidney tissues was determined spectrophotometrically as described in earlier literature [1]. The MPO activity was expressed as U/g of tissue.

### Preparation of total, nuclear and cytoplasmic protein extracts

Radioimmunoprecipitation (RIPA) lysis buffer and NE-PER nuclear and cytoplasmic extraction kit containing 1% Halt protease inhibitor cocktail (Pierce Biotechnology, Rockford, IL, USA) were used to isolate the total, nuclear and cytoplasmic fraction from the kidney tissues as described in manufacturer instructions. The protein concentration in each fraction was determined by using Bicinchoninic acid (BCA) protein assay kit (Pierce Biotechnology, Rockford, IL, USA) against bovine serum albumin (BSA) as standard.

### Western blot analysis

Western blot was carried out as described previously [1]. In total protein extract, expressions of Bax (rabbit, monoclonal, Cell Signaling Technology; 1:1000), Bcl-2 (rabbit, monoclonal, Cell Signaling Technology; 1:1000), cleaved caspase-3 (rabbit, monoclonal, Cell Signaling Technology; 1:1000), cleaved caspase-9 (mouse, monoclonal, Cell Signaling Technology; 1:1000), cleaved PARP (rabbit, monoclonal, Cell Signaling Technology; 1:1000), p53 (mouse, monoclonal, Cell Signaling Technology; 1:1000), iNOS (rabbit, monoclonal, Sigma-Aldrich; 1:500), Nrf2 (rabbit, monoclonal, Cell Signaling Technology; 1:500), HO-1 (rabbit, monoclonal, Cell Signaling Technology; 1:1000), ERK1/2 (rabbit, monoclonal, Pierce Biotechnology, 1:1000), phospho-ERK1/2 (rabbit, monoclonal, Pierce Biotechnology, 1:1000), p38 (rabbit, monoclonal, Pierce Biotechnology, 1:1000), phospho-p38 (rabbit, monoclonal, Pierce Biotechnology, 1:1000), JNK (rabbit, monoclonal, Pierce Biotechnology, 1:1000), phospho-JNK (rabbit, monoclonal, Pierce Biotechnology, 1:1000); in mitochondrial and cytosolic fractions, expression of cytochrome c (rabbit, monoclonal, Cell Signaling Technology; 1:1000); in nuclear fractions, expression of Nrf2 (rabbit, monoclonal, Cell Signaling Technology; 1:500) and NF-κB (p65) (rabbit, monoclonal, Cell Signaling Technology; 1:500) and in cytoplasmic fraction, the expressions of Nrf2 (rabbit, monoclonal, Cell Signaling Technology; 1:500), NF-κB (p65) (rabbit, monoclonal, Cell Signaling Technology; 1:500), phospho-IKKα/β (rabbit, monoclonal, Cell Signaling Technology; 1:500), phospho-IκBα (rabbit, monoclonal, Cell Signaling Technology; 1:1000) and IκBα (mouse, monoclonal, Cell Signaling Technology; 1:1000) were estimated. The immunoreactive bands were visualized with chemiluminescent detection reagents (Supersignal West Pico, Pierce Biotechnology, Rockford, IL, USA) and Vilber-Fusion-Western blot-Chemiluminescence Imaging system. The densitometry analysis of each blot was performed by employing Image J software, NIH, USA.

### NF-κB (p65)-DNA binding assay

The nuclear protein fraction from each experimental animal was used for NF-κB-DNA binding assay. The NF-κB-DNA binding activity was assessed by using a commercial available NF-κB (p65) transcription factor ELISA assay kit (Cayman Chemical Company, Ann Arbor, MI) according to the manufacturer’s protocol.

### Statistical Analysis

The statistical analyses were performed using Graph Pad Prism software (version 5.0). All of the values were presented as mean ± standard error of mean (SEM) and differences between groups were compared with one-way ANOVA followed by Dunnett's multiple comparison procedure. A p< 0.05 was regarded statistical significant.

## Results

### Baicalein ameliorates cisplatin-induced acute renal injury

**Fig 1:**
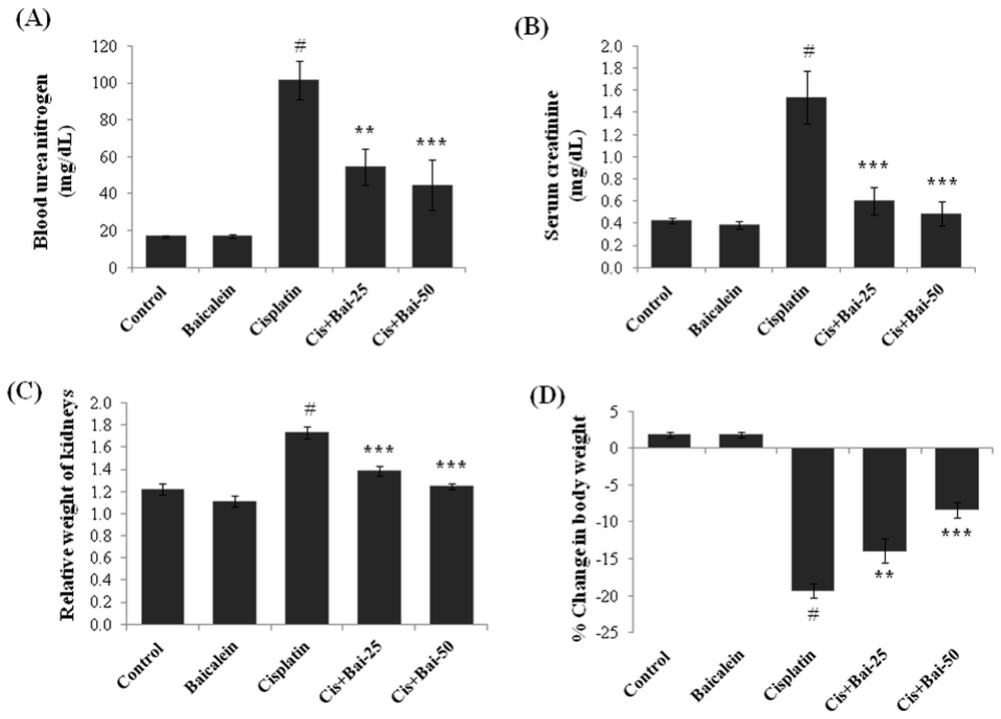
Effect of baicalein on cisplatin-induced renal injury indicators. (A) Blood urea nitrogen (BUN), (B) Creatinine, (C) Relative weight of kidneys and (D) % change in body weight. Values are the means ± SEM (n = 8). Where Control, group of animals treated with vehicle (2% gum acacia suspension, orally) daily for 15 consecutive days; Baicalein, group of animals treated with baicalein (50 mg/kg baicalein in 2% gum acacia suspension, orally) daily for 15 consecutive days; Cisplatin, group of animals treated with 2% gum acacia suspension (orally) daily for 15 consecutive days and a single intraperitoneal (i.p) injection of cisplatin (20 mg/kg body weight, dissolved in normal saline) on 12^th^ day; Cis+Bai-25, group of animals treated with baicalein (25 mg/kg, orally) daily for 15 consecutive days and a single intraperitoneal (i.p) injection of cisplatin (20 mg/kg body weight, dissolved in normal saline) on 12^th^ day; Cis+Bai-50, group of animals treated with baicalein (50 mg/kg, orally) daily for 15 consecutive days and a single intraperitoneal (i.p) injection of cisplatin (20 mg/kg body weight, dissolved in normal saline) on 12^th^ day. ^#^p< 0.001 vs. vehicle control group, **p< 0.01 and ***p< 0.001 vs. cisplatin control group.

As shown in Fig 1, the cisplatin alone treated mice demonstrated a marked deterioration of their renal function 72h after the cisplatin injection. The serum levels of renal injury biomarkers, such as BUN (Fig 1A) and creatinine (Fig 1B) and the relative weight of kidneys (Fig 1C) were significantly (p< 0.001) increased and the body weight (Fig 1D) was significantly (p< 0.001) decreased in cisplatin alone administered mice when compared with the vehicle control mice. HoPretreatment with baicalein at both the doses (25 and 50 mg/kg) followed by cisplatin significantly attenuated the elevation of BUN (p< 0.01 at 25mg/kg and p< 0.001 at 50 mg/kg baicalein) and serum creatinine levels (p< 0.001 at both 25 and 50 mg/kg baicalein) and prevented the increase in relative weight of kidneys (p< 0.001 at both 25 and 50 mg/kg baicalein) and the body weight loss (p< 0.01 at 25mg/kg and p< 0.001 at 50 mg/kg baicalein) compared with the cisplatin alone administered mice.

**Fig 2:**
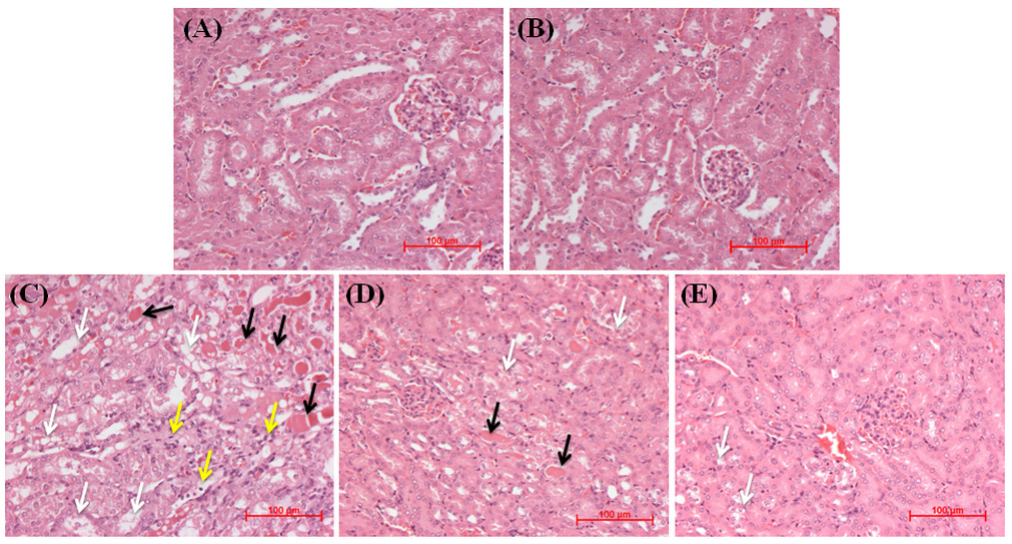
Representative photomicrographs and light microscopic examination (H and E staining) of kidney tissues treated with cisplatin and/or baicalein. Kidney tissue sections from control (A) and baicalein alone (B) treated mice showing normal kidney architecture. Kidney tissue sections from mice treated with cisplatin alone (C) showing obvious structural damage such as tubular degeneration, extensive epithelial vacuolization and necrosis (white arrow), massive infiltration of inflammatory cells (yellow arrow) and formation of hyaline casts (black arrow) in the renal tubules. Mice pretreated with baicalein at 25 mg/kg followed by cisplatin (D) showing mild to moderate degenerative changes (white arrow) with occasional casts (black arrow). Mice pretreated with baicalein at 50 mg/kg followed by cisplatin (E) showing apparently normal glomerular and tubular structure with mild tubular degenerative changes (white arrow) and without infiltration of inflammatory cells and tubular casts in the lumen.

Corroborating with the functional analysis, hematoxylin and eosin (H & E)-stained kidney tissue sections (Fig 2) from either vehicle control (Fig 2A) or baicalein alone (Fig 2B) treated mice showed apparently normal kidney architecture. Kidney tissue sections from mice treated with cisplatin alone (Fig 2C) revealed obvious structural damage such as tubular degeneration, extensive epithelial vacuolization and necrosis, massive infiltration of inflammatory cells and formation of hyaline casts in the renal tubules. Mice pretreated with baicalein at 25 mg/kg followed by cisplatin (Fig 2D) showed lesser tissue damage with occasional casts compared with the cisplatin alone treated mice. However, pretreatment with baicalein at 50 mg/kg followed by cisplatin (Fig 2E) markedly attenuated the histopathological changes and prevented the infiltration of inflammatory cells and formation of casts in the renal tubules.

### Baicalein restores cisplatin-induced decline in renal antioxidant defense

As shown in Table 1, cisplatin administration caused a significant suppression of the renal antioxidant defense, as observed by a decrease in the GSH (p< 0.01) and Vit C (p< 0.001) levels and in GR (p< 0.001), GST (p< 0.01), SOD (p< 0.001), CAT (p< 0.001) and NQO1 (p< 0.001) activities compared with the vehicle control mice. Pretreatment with baicalein at 50 mg/kg significantly (p< 0.05 for NQO1; p< 0.01 for GSH, SOD and CAT; p< 0.001 for GR, GST and Vit C) restored these changes to near normal range when compared with cisplatin alone treated mice. Though baicalein pretreatment at 25mg/kg significantly attenuated the decrease in renal antioxidant levels and/or activities, such as GR (p< 0.01), GST (p< 0.05), SOD (p< 0.01) and vitamin C (p< 0.001), the levels of GSH and the activities of CAT and NQO1 were remained unaltered (p> 0.05) when compared with the cisplatin alone treated mice.

**Table 1:** Effect of baicalein on cisplatin-induced alterations in renal antioxidants.

	Control	Baicalein	Cisplatin	Cis+Bai-25	Cis+Bai-50
GSH levels	0.171 ±0.01	0.184 ± 0.01	0.095 ± 0.01^†^	0.124 ± 0.01	0.172 ± 0.02**
GR activity	1.897 ± 0.30	2.012 ± 0.05	0.681 ± 0.11^#^	1.682 ± 0.23**	1.992 ± 0.14***
GST activity	17.495 ± 1.41	19.019 ± 1.03	10.278 ± 0.83^†^	15.408 ± 0.82*	23.791 ± 2.00***
SOD activity	100.000 ± 1.57	103.980 ± 0.62	70.311 ± 1.28^#^	81.244 ± 2.45**	83.205 ± 0.48**
CAT activity	11.591 ± 0.47	11.237 ± 0.51	8.123 ± 0.38^#^	8.843 ± 0.27	10.175 ± 0.47**
NQO1 activity	55.357 ± 6.75	68.452 ± 2.44	24.901 ± 0.64^#^	38.180 ± 4.12	46.825 ± 3.66*
Vitamin C levels	0.512 ± 0.03	0.545 ± 0.03	0.112 ± 0.02^#^	0.345 ± 0.02***	0.458 ± 0.02***

All data were expressed as mean ± S.E.M, N = 6. Where, Control, vehicle (2% gum acacia) treated mice; Baicalein, baicalein (50mg/kg) alone treated mice; Cisplatin, cisplatin-induced mice; Cis+Bai-25, baicalein at a dose of 25 mg/kg body weight treated cisplatin-induced mice; Cis+Bai-50, baicalein at a dose of 50 mg/kg body weight treated cisplatin-induced mice; GSH, reduced glutathione (mg/g tissue); GR, glutathione reductase (U/mg protein); GST, glutathione S-transferase (nmol of CDNB conjugated/ min/ mg/ protein); SOD, superoxide dismutase (% of control); CAT, catalase (U/mg protein); NQO1, NAD(P)H: quinone oxidoreductase 1 (nmol of DCIP reduced/min/ mg/ protein); CDNB, 1-chloro-2, 4-dinitrobenzene; DCIP, 2, 6-dichlorophenolindophenol.

^#^p< 0.001 and ^†^p< 0.01 vs Vehicle control,

*p< 0.05, **p< 0.01 and ***p< 0.001 vs Cisplatin control group.

### Baicalein enhances nuclear translocation of Nrf2 and increases HO-1 expression in kidney tissues

**Fig 3:**
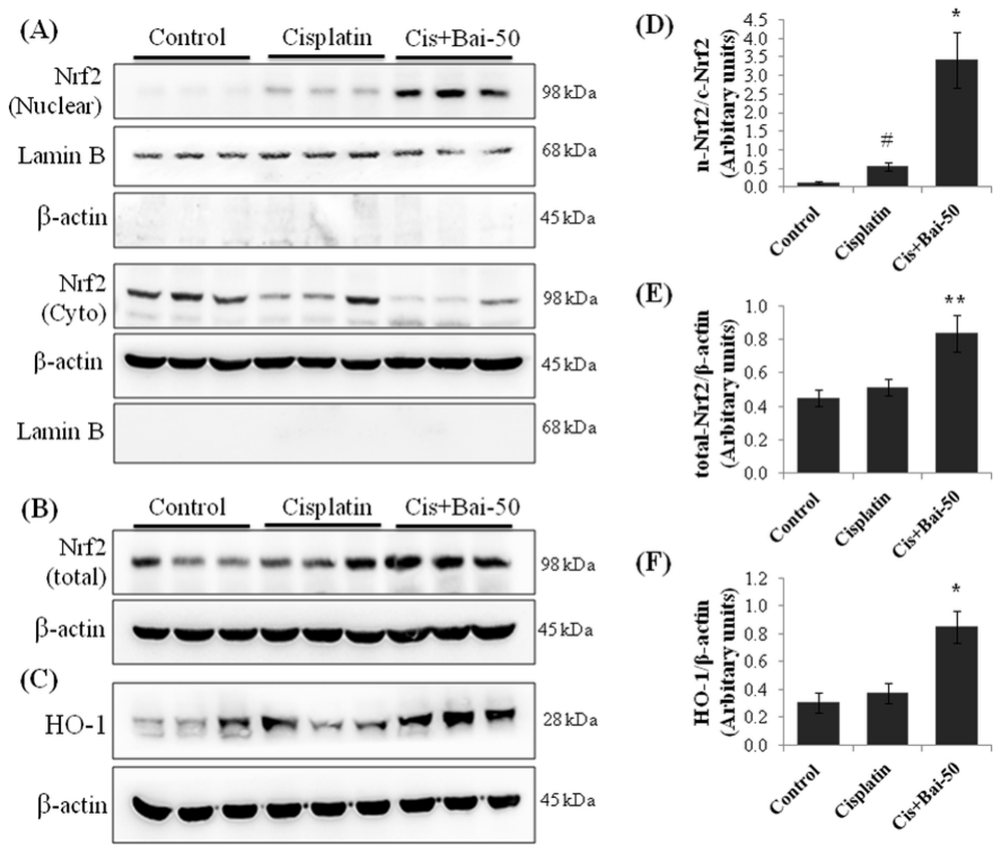
Effect of baicalein and/or cisplatin on nuclear, cytoplasmic and total Nrf2 and the HO-1 expression. Immunoblot analyses showing (A) nuclear translocation of Nrf2, (B) total Nrf2 and (C) HO-1 in kidneys. Immunoblots were representative of three independent experiments. Lamin B was used as internal control for nuclear fraction and β-actin was used as internal control for cytoplasmic and total protein fractions. Bar diagram showing densitometric analysis for relative expression of (D) nuclear Nrf2/cytoplasmic Nrf2 ratio, (E) total Nrf2 and (F) HO-1 proteins. Values are the means ± SEM (n = 3). Where Control, group of animals treated with vehicle (2% gum acacia suspension, orally) daily for 15 consecutive days; Cisplatin, group of animals treated with 2% gum acacia suspension (orally) daily for 15 consecutive days and a single intraperitoneal (i.p) injection of cisplatin (20 mg/kg body weight, dissolved in normal saline) on 12^th^ day; Cis+Bai-50, group of animals treated with baicalein (50 mg/kg, orally) daily for 15 consecutive days and a single intraperitoneal (i.p) injection of cisplatin (20 mg/kg body weight, dissolved in normal saline) on 12^th^ day; Nrf2, nuclear factor erythroid 2-related factor 2; HO-1, heme oxygenase-1. ^#^p< 0.05 vs. vehicle control group, *p< 0.05 and **p< 0.01 vs. cisplatin control group.

To determine whether baicalein treatment could influence the Nrf2 activation, we assessed the nuclear translocation of Nrf2 by determining the amount of Nrf2 protein in the nuclear, cytoplasmic and total protein extracts in kidney tissues (Fig 3). There was increased (p< 0.05) nuclear accumulation of Nrf2 protein in kidneys of cisplatin alone treated mice compared with the vehicle control mice. Pretreatment with baicalein at 50 mg/kg further intensified (p< 0.05) the nuclear accumulation of Nrf2 when compared with the cisplatin alone treated mice (Fig 3A). Furthermore, the amount of Nrf2 (Fig 3B) and HO-1 (Fig 3C) expression in the total protein extracts of kidney tissues from mice pretreated with baicalein at 50 mg/kg followed by cisplatin were significantly (p< 0.01 for Nrf2; p< 0.05 for HO-1) increased when compared with the cisplatin alone treated mice. However, in total protein extracts, we have not observed any significant difference (p> 0.05) in the amount of Nrf2 and HO-1 expressions between the vehicle control and cisplatin alone treated groups.

### Baicalein attenuates cisplatin-induced renal lipid peroxidation and nitrative stress

**Fig 4:**
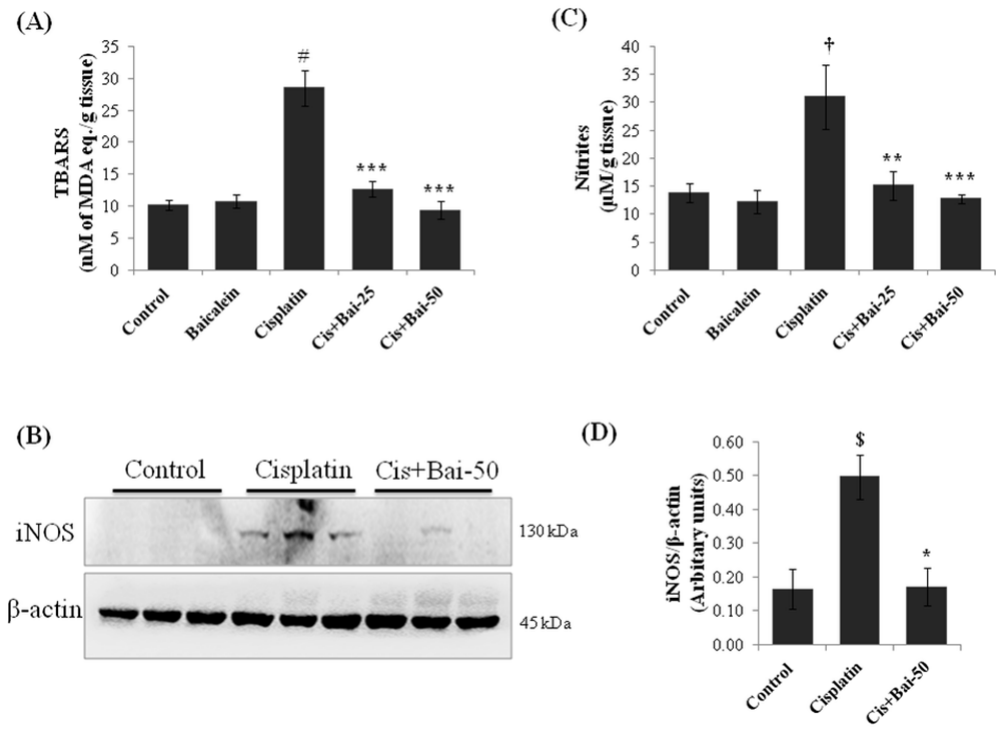
Effect of baicalein on cisplatin-induced renal lipid peroxidation and nitrative stress. (A) Thiobarbituric acid reactive substances (TBARS), as a marker for lipid peroxidation, (B) Immunoblot of iNOS and (C) tissue nitrate levels, as markers for nitrative stress. Immunoblot was representative of three independent experiments. β-actin was used as internal control. Bar diagram showing densitometric analysis for relative expression of (D) iNOS and values are the means ± SEM (n = 3). For the estimation of TBARS and nitrites, the data were expressed as the means ± SEM of 8 animals. Where iNOS, inducible nitric oxide synthase; MDA, malondialdehyde. ^$^p< 0.05, ^†^p< 0.01 and ^#^p< 0.001 vs. vehicle control group, *p< 0.05, **p< 0.01 and ***p< 0.001 vs. cisplatin control group.

As shown in Fig 4, there was increased (p< 0.001) accumulation of thiobarbituric acid reactive substances (TBARS) (Fig 4A), as an index of lipid peroxidation and expression of inducible nitric oxide synthase (iNOS) (Fig 4B) with concordant increase in tissue nitrite levels (Fig 4C), as a marker of nitrative stress (p< 0.05 for iNOS and p< 0.01 for nitrites) in kidneys of cisplatin alone treated mice when compared with vehicle control mice. Conversely, pretreatment with baicalein significantly reduced the TBARS (p< 0.001 at both 25 and 50 mg/kg baicalein) and nitrite levels (p< 0.01 at 25mg/kg and p< 0.001 at 50 mg/kg baicalein) and attenuated the iNOS expression (p< 0.05 at 50mg/kg) when compared with the cisplatin alone treated mice.

### Baicalein restores cisplatin-induced decline in mitochondrial respiratory and MnSOD enzyme activities and inhibits cytochrome c release

**Fig 5:**
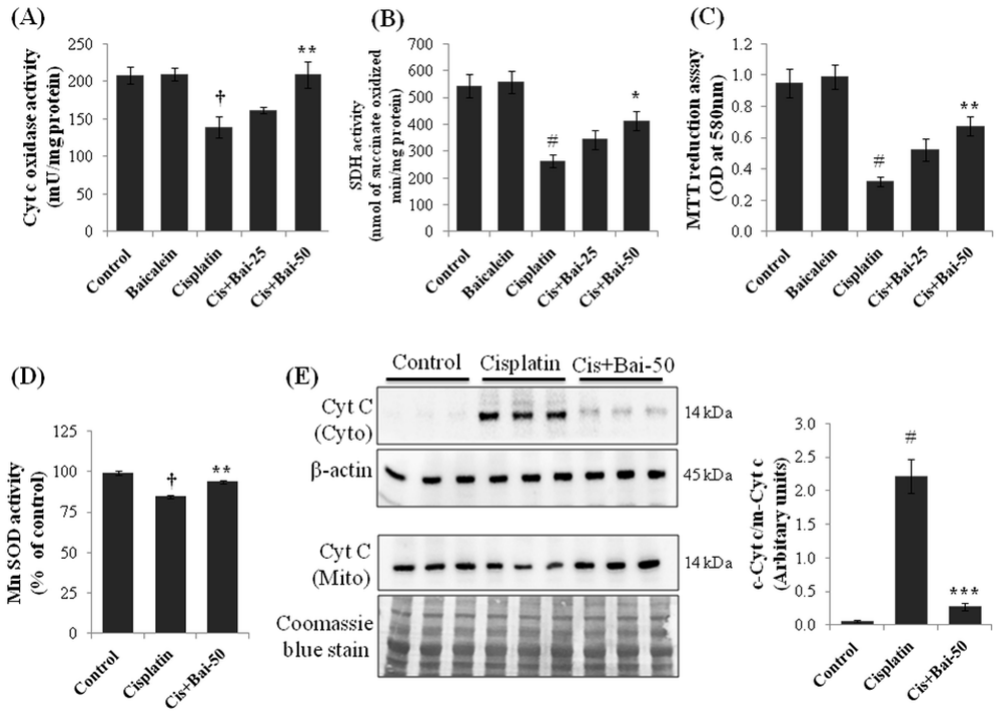
Effect of baicalein on cisplatin-induced impairment of mitochondrial respiratory and MnSOD enzyme activities and cytochrome c release. Pretreatment with baicalein restored cisplatin-induced decline in mitochondrial (A) Cytochrome c oxidase (COX), (B) Succinate dehydrogenase (SDH), (C) Redox (MTT reduction) and (D) MnSOD activities in kidney tissues. The data were expressed as the means ± SEM of 8 animals. (E) Immunoblot analyses and bar diagram showing densitometric analysis of cytosolic translocation of cytochrome c (Cyt c) from mitochondria. Values are the means ± SEM (n = 3). Immunoblots were representative of three independent experiments. β-actin was used as internal control for cytosolic fractions. Coomassie blue stain was used for equal loading of mitochondrial fractions. ^†^p< 0.01 and ^#^p< 0.001 vs. vehicle control group, *p< 0.05, **p< 0.01 and ***p< 0.01 vs. cisplatin control group.

Mitochondrial respiratory enzymes, such as the activities of cytochrome c oxidase (COX) (Fig 5A) and succinate dehydrogenase (SDH) (Fig 5B) and the mitochondrial redox activity (MTT reduction assay, as marker of mitochondrial intactness) (Fig 5C) were significantly (p< 0.01 for COX; p< 0.001 for SDH and MTT) decreased in mice exposed to only cisplatin when compared with the vehicle control group of mice. Conversely, pretreatment with baicalein at 50 mg/kg significantly (p< 0.01 for COX; p< 0.05 for SDH) prevented the depletion of COX and SDH and restored these enzyme activities to their normal value. In addition, mitochondrial redox activity was also significantly (p< 0.01) increased in the baicalein (50 mg/kg) pretreated cisplatin-induced mice compared with the cisplatin alone treated mice. Pretreatment with baicalein at 25 mg/kg did not produce any significant (p> 0.05) change in these activities compared with the cisplatin alone treated mice. Mitochondrial specific SOD enzyme, manganese superoxide dismutase (MnSOD) is one of the key enzymes which keep in check the levels of excess mitochondrial superoxide and oxidative stress. As shown in Fig 5D, the activity of MnSOD was significantly (p< 0.01) decreased in the kidney tissues of cisplatin alone treated mice when compared with the vehicle control mice. Pretreatment with baicalein (50 mg/kg) significantly (p< 0.01) restored the cisplatin-induced decrease in MnSOD activity in kidney tissues when compared with the cisplatin alone treated mice. We next examined the role of cytochrome c and its cytosolic translocation, a critical event in kidney cell apoptosis during cisplatin exposure. As shown in Fig 5E, in cisplatin-induced mice, a significant (p< 0.001) amount of mitochondrial cytochrome c moved to the cytosolic fraction when compared with the vehicle control. Pretreatment with baicalein (50 mg/kg) effectively (p< 0.001) suppressed the cisplatin-induced mitochondrial cytochrome c release in to the cytosol.

### Baicalein attenuates cisplatin-induced apoptotic-related protein expression

**Fig 6:**
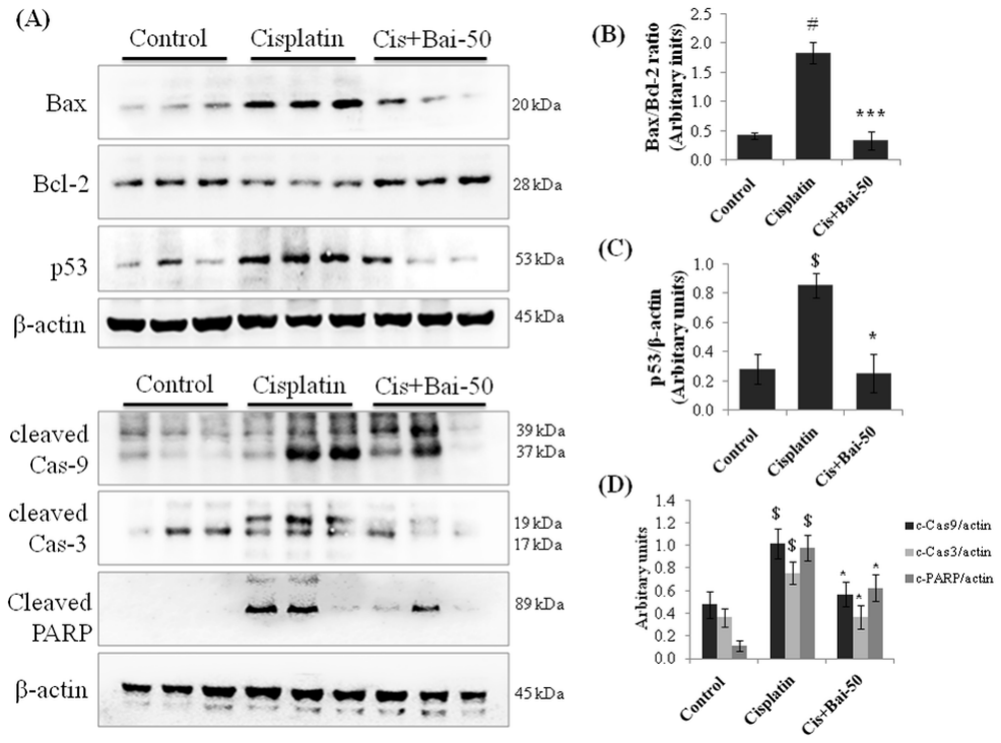
Effect of baicalein on cisplatin-induced renal apoptotic proteins. Immunoblot analyses showing expression levels of Bax, Bcl-2, p53, cleaved caspase-9, cleaved caspase-3 and cleaved PARP in kidneys (A). Immunoblots were representative of three independent experiments. β-actin was used as internal control. Bar diagram showing densitometric analysis for relative expression of (B) Bax/Bcl-2 ratio, (C) p53 and (D) cleaved caspase-9, 3 and PARP. Values are the means ± SEM (n = 3). Where Control, group of animals treated with vehicle (2% gum acacia suspension, orally) daily for 15 consecutive days; Cisplatin, group of animals treated with 2% gum acacia suspension (orally) daily for 15 consecutive days and a single intraperitoneal (i.p) injection of cisplatin (20 mg/kg body weight, dissolved in normal saline) on 12^th^ day; Cis+Bai-50, group of animals treated with baicalein (50 mg/kg, orally) daily for 15 consecutive days and a single intraperitoneal (i.p) injection of cisplatin (20 mg/kg body weight, dissolved in normal saline) on 12th day; PARP, poly (ADP-ribose) polymerase. ^$^p< 0.05 and ^#^p< 0.01 vs. vehicle control group, *p< 0.05 and ***p< 0.001 vs. cisplatin control group.

As shown in Fig 6, the amounts of Bax/Bcl-2 ratio, p53, cleaved caspase-9, cleaved caspase-3 and PARP expressions were significantly (p< 0.01 for Bax/Bcl-2 ratio; p< 0.05 for p53) increased in cisplatin alone administered mice when compared with the vehicle control mice. Pretreatment with baicalein blocked the increase in cisplatin-induced Bax/Bcl-2 ratio (p< 0.001) and p53 (p< 0.05) expression and ameliorated the caspase-9, -3 and PARP activation in kidney tissues when compared with the cisplatin alone treated mice.

### Baicalein inhibits cisplatin-induced NF-κB (p65) nuclear translocation, NF-κB-DNA binding and IκBα degradation

**Fig 7:**
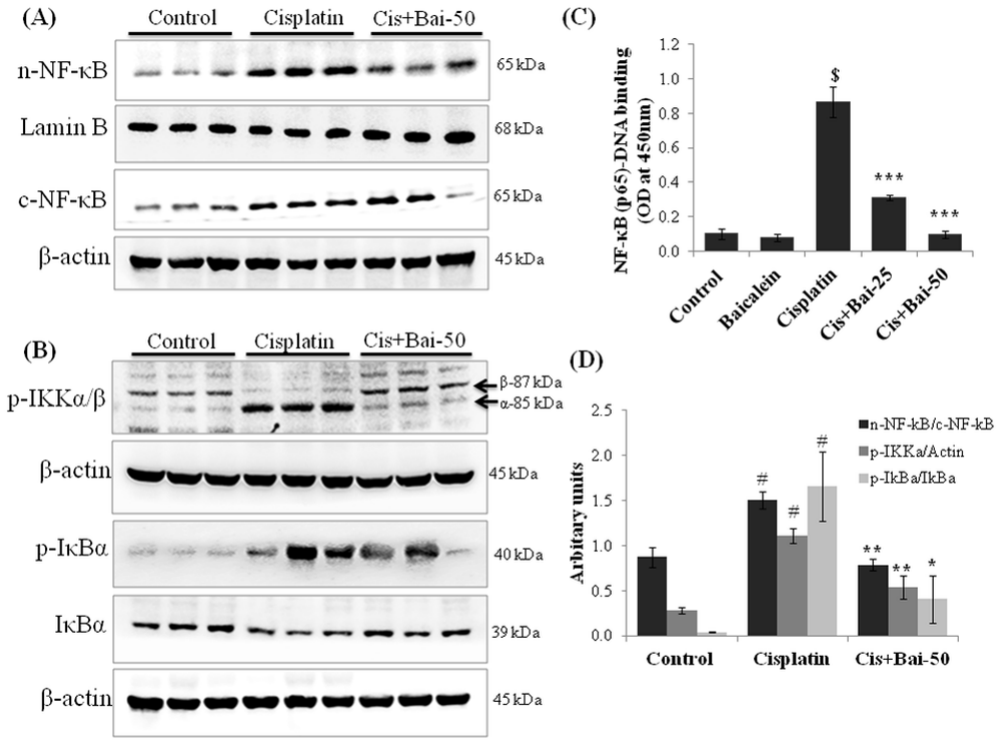
Effect of baicalein on cisplatin-induced NF-κB related proteins. Immunoblot analyses showing (A) nuclear translocation of NF-κB (p65), (B) phospho-IKKα/β, phospho-IκBα and IκBα expressions in kidneys. (C) NF-κB (p65)-DNA binding activity, where values are expressed as means ± SEM (n = 6). Immunoblots were representative of three independent experiments. Lamin B was used as internal control for nuclear fraction and β-actin was used as internal control for cytoplasmic and total protein fractions. Bar diagram showing densitometric analysis for relative expression of (D) nuclear NF-κB (p65)/cytoplasmic NF-κB (p65) ratio, phospho-IKKα/β-actin and phospho-IκBα/IκBα ratio. Values are the means ± SEM (n = 3). Where Control, group of animals treated with vehicle (2% gum acacia suspension, orally) daily for 15 consecutive days; Cisplatin, group of animals treated with 2% gum acacia suspension (orally) daily for 15 consecutive days and a single intraperitoneal (i.p) injection of cisplatin (20 mg/kg body weight, dissolved in normal saline) on 12^th^ day; Cis+Bai-50, group of animals treated with baicalein (50 mg/kg, orally) daily for 15 consecutive days and a single intraperitoneal (i.p) injection of cisplatin (20 mg/kg body weight, dissolved in normal saline) on 12^th^ day; ^#^p< 0.05 and ^$^p< 0.001 vs. vehicle control group, *p< 0.05, **p< 0.01 and ***p< 0.001 vs. cisplatin control group.

As shown in Fig 7, there was a significant (p< 0.05) increase in the NF-κB (p65) accumulation in the nuclear fractions of the kidney tissues from the cisplatin-induced mice when compared with the vehicle control mice (Fig 7A). In addition, there was a marked (p< 0.05) phosphorylation of IKKα/β protein with subsequent degradation and phosphorylation of IκBα (p< 0.05 for p-IκBα/IκBα ratio) in the cytoplasmic fraction of kidney tissues from the cisplatin-induced mice when compared with the vehicle control mice (Fig 7B). NF-κB (p65) transcription factor ELISA assay also confirmed the enhanced (p< 0.001) DNA-binding activity of p65 subunit of NF-κB in the nuclear fractions of the kidney tissues from the cisplatin-induced mice (Fig 7C). The baicalein (50 mg/kg) pretreatment significantly prevented the cisplatin-induced IKKα/β and IκBα phosphorylation, IκBα degradation and subsequent nuclear translocation and DNA binding activity of NF-κB (p65) when compared with the cisplatin alone treated mice.

### Baicalein attenuates cisplatin-induced renal inflammation

**Fig 8:**
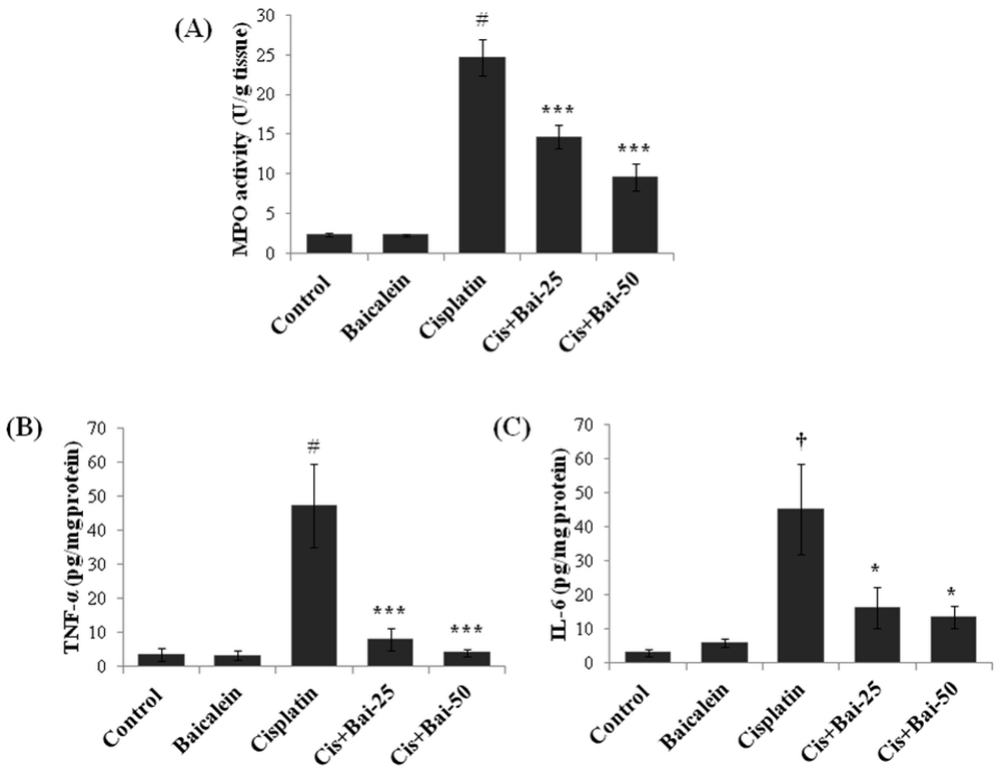
Effect of baicalein on cisplatin-induced renal inflammatory mediators. (A) Myeloperoxidase (MPO) activity, (B) Tumor necrosis factor- α (TNF-α) and (C) Interleukin-6. Values are the means ± SEM (n = 8). Where Control, group of animals treated with vehicle (2% gum acacia suspension, orally) daily for 15 consecutive days; Baicalein, group of animals treated with baicalein (50 mg/kg baicalein in 2% gum acacia suspension, orally) daily for 15 consecutive days; Cisplatin, group of animals treated with 2% gum acacia suspension (orally) daily for 15 consecutive days and a single intraperitoneal (i.p) injection of cisplatin (20 mg/kg body weight, dissolved in normal saline) on 12^th^ day; Cis+Bai-25, group of animals treated with baicalein (25 mg/kg, orally) daily for 15 consecutive days and a single intraperitoneal (i.p) injection of cisplatin (20 mg/kg body weight, dissolved in normal saline) on 12^th^ day; Cis+Bai-50, group of animals treated with baicalein (50 mg/kg, orally) daily for 15 consecutive days and a single intraperitoneal (i.p) injection of cisplatin (20 mg/kg body weight, dissolved in normal saline) on 12^th^ day. ^†^p< 0.01 and ^#^p< 0.001 vs. vehicle control group, *p< 0.05 and ***p< 0.001 vs. cisplatin control group.

The myeloperoxidase (MPO) activity (Fig 8A) and the levels of pro-inflammatory cytokines, TNF-α (Fig 8B) and IL-6 (Fig 8C) were markedly (p< 0.001 for TNF-α and MPO; p< 0.01 for IL-6) elevated in mice exposed to cisplatin when compared with the vehicle control mice. Baicalein pretreatment (both 25 and 50 mg/kg) significantly (p<0.001 for TNF-α and MPO; p< 0.05 for IL-6) attenuated these elevations when compared with the cisplatin alone treated mice.

### Baicalein inhibits MAPK pathways

**Fig 9:**
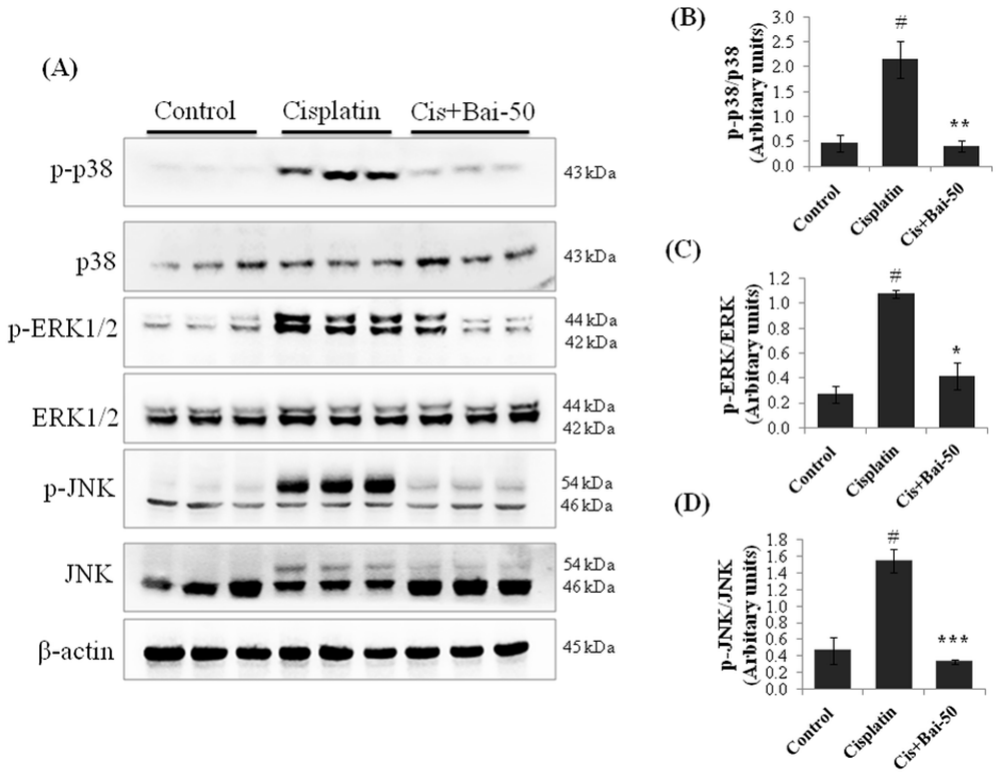
Effect of baicalein on cisplatin-induced MAPKs activation. Immunoblot analyses showing expression levels of phospho-p38, p38, phospho-ERK1/2, ERK1/2, phospho-JNK and JNK (A). Immunoblots were representative of three independent experiments. β-actin was used as internal control. Bar diagram showing densitometric analysis for relative expression of (B) p-p38/p38, (C) p-ERK/ERK and (D) p-JNK/JNK. Values are the means ± SEM (n = 3). Where Control, group of animals treated with vehicle (2% gum acacia suspension, orally) daily for 15 consecutive days; Cisplatin, group of animals treated with 2% gum acacia suspension (orally) daily for 15 consecutive days and a single intraperitoneal (i.p) injection of cisplatin (20 mg/kg body weight, dissolved in normal saline) on 12^th^ day; Cis+Bai-50, group of animals treated with baicalein (50 mg/kg, orally) daily for 15 consecutive days and a single intraperitoneal (i.p) injection of cisplatin (20 mg/kg body weight, dissolved in normal saline) on 12^th^ day. ^#^p< 0.01 vs. vehicle control group, *p< 0.05, **p< 0.01 and ***p< 0.001 vs. cisplatin control group.

We subsequently investigated the possible effects of baicalein and cisplatin on MAPK activation by western blot analysis. As shown in Fig 9, there was marked increase in the phosphorylation of p38 (p< 0.01), ERK1/2 (p< 0.01) and JNK (p< 0.01) in the kidneys of cisplatin-induced mice. Interestingly, pretreatment with baicalein (50 mg/kg) significantly (p< 0.01 for p-p38, p< 0.05 for p-ERK and p< 0.001 for p-JNK) ameliorated all these changes when compared with the cisplatin alone treated mice.

## Discussion

Acute kidney injury is a serious complication of the anticancer drug cisplatin [38, 39]. We demonstrated that baicalein, a bioflavonoid with potent antioxidant and anti-inflammatory properties effectively ameliorates the cisplatin-induced renal impairment in mice. Reduced oxidative and/or nitrative stress, improved mitochondrial function and decreased inflammation and renal tubular cell death in kidneys of baicalein pre-treated cisplatin-induced animals emphasize the apparent beneficial effect of baicalein in the contest of cisplatin-induced nephrotoxicity. Increased production of ROS and subsequent disturbance in antioxidant defense in kidneys has been widely reported in cisplatin-induced animals [25, 40]. Excess generation of ROS attacks endogenous intracellular targets such as DNA, lipids and proteins and cause modification of these to exhibit cellular dysfunction and apoptosis [38]. Moreover, cisplatin-induced ROS activates numerous signaling pathways including MAPKs, NF-κB, and p53 and exacerbate its toxic effects [25]. The expression of iNOS and the formation of potent cytotoxic peroxynitrite through the interaction of superoxide radical with nitric oxide also play an important role in the pathogenesis of cisplatin-induced renal injury [40]. Baicalein has been shown to have a potent antioxidant effect [13]. It has been reported that baicalein effectively scavenges peroxynitrite anion radical and also prevents peroxynitrite-induced cell death in LLC-PK_1_ cells [41]. Result of the present study showed that cisplatin notably decreased the levels and/or activities of both enzymatic and non-enzymatic antioxidants and impaired antioxidant defense mechanisms in the kidneys. Moreover, the lipid peroxidation marker, TBARS and tissue levels of nitrites and iNOS expression were also significantly increased in cisplatin administered mice. Pretreatment with baicalein significantly attenuated these changes. Therefore, it is likely that the reduction of cisplatin-induced nephrotoxicity by baicalein is, at least in part, due to the attenuation of renal oxidative and/or nitrative stress.

The transcription factor, Nrf2 is known to be a main defense mechanism against oxidative stress in cells. Previous studies have investigated the role of Nrf2, and specifically up-regulation of Nrf2 as a strategy to prevent the cisplatin-induced nephrotoxicity [11, 12]. Induction of antioxidant defense and phase II detoxifying enzymes has been positively associated with Nrf2 expression and its nuclear translocation [42]. To explore the effect of baicalein pretreatment on renal Nrf2 expression and its role in cisplatin-induced renal injury, we assessed the nuclear translocation of Nrf2 and the expression of HO-1 by western blotting. Cisplatin administration induced a significant amount of nuclear Nrf2 in kidneys compared with the vehicle control mice. We suggest that nuclear accumulation of Nrf2 in cisplatin alone treated mice is not specific to cisplatin administration as Nrf2 nuclear levels were also increased due to other oxidative stress related stimuli, such as hydrogen peroxide, ischemia and exercise [43]. However, pretreatment with baicalein further intensified the nuclear accumulation of Nrf2 when compared with the cisplatin alone treated mice. In corroborating to our findings, it has been demonstrated that baicalein can modulate Nrf2/Keap1 pathway through both Keap1-independent mechanism by targeting Akt, and JNK1/2 upstream pathways and Keap1-dependent mechanism by increasing Keap1 ubiquitination and subsequent nuclear translocation in HepG2 cells [44]. Heme oxygenase 1 (HO-1), an inducible cytoprotective enzyme, is the most well-known downstream genes of Nrf2 and is involved in the regulation of intracellular redox-balancing [8]. The amount of renal HO-1 expression in the baicalein pretreated cisplatin-induced mice was significantly increased when compared with the cisplatin alone treated mice. Hence, we believe that upregulation of Nrf2 expression and its downstream cytoprotective protein, HO-1 due to baicalein administration is, at least in part, may account for its antioxidant defense mechanisms against cisplatin-induced oxidative damage in kidneys.

A growing body of evidence suggests that mitochondrion is one of the subcellular targets of cisplatin and plays an important role in the induction of renal damage [45]. Mitochondria, whose main function is to produce energy by oxidative phosphorylation, are key endogenous sources of ROS [46]. Oxidative damage of mitochondria and the impairment of mitochondrial respiratory enzyme activities have been implicated in the pathogenesis of cisplatin-induced nephrotoxicity [47, 48]. Mitochondria-localized manganese superoxide dismutase (MnSOD) scavenges the level of excess mitochondrial superoxide and involved in cisplatin-induced nephroprotection [48, 49]. In the present study, we assessed the effect of baicalein on mitochondrial respiratory function by measuring cytochrome c oxidase (COX), succinate dehydrogenase (SDH) and mitochondrial redox activity and the mitochondrial antioxidant defense by measuring MnSOD activity. As expected, a reduction in the activity of COX, SDH and redox activity of the respiratory chain and the MnSOD activity following cisplatin administration is clearly indicative of mitochondrial dysfunction. Treatment with baicalein (50 mg/kg) improved these activities to normal level and protected mitochondria from cisplatin-induced damage.

Cisplatin-induced renal cell death involves multiple pathways [39]. In kidneys, cisplatin alters mitochondrial membrane potential, activates Bax, reduces Bcl-2 and shifts the Bax/Bcl-2 ratio in a pro-apoptotic direction [3]. The imbalance between pro-apoptotic and antiapoptotic force cause permeabilisation of mitochondrial membrane, cytochrome c release and ultimately apoptosis through caspases activation. In the present study, we found that pretreatment with baicalein prevented the mitochondrial cytochrome c release and attenuated the activation of apoptotic pathways by restoring the aforementioned proteins. The role of p53, a proapoptotic protein and its related signaling pathway has been associated with cisplatin-induced nephrotoxicity [25, 35]. Moreover, it has been demonstrated that p53-deficient mice are resistant to cisplatin-elicited kidney injury [35]. It has also been demonstrated that p53 induction can induce apoptosis through the direct activation of pro-apoptotic protein, Bax [39]. In this experiment, we demonstrated that pretreatment with baicalein decreased the cisplatin-elevated p53 expression in kidney tissues. We therefore suggest that baicalein decreases cisplatin-induced cell death through the regulation of both p53 and the mitochondrial-dependent intrinsic apoptosis pathways in kidney tissues.

Inflammation plays an important role in the initiation and progression of cisplatin-induced renal damage [25, 26]. Cisplatin induces release of a series of proinflammatory cytokines (TNF-α, IL-1β and IL-6) and causes the infiltration of leukocytes and macrophages within 72h in damaged renal tissues [6]. It has also been demonstrated that pharmacological inhibition or genetic deletion of TNF-α, reduces cisplatin-induced epithelial cell necrosis and apoptosis, infiltration of leukocytes and renal dysfunction [26]. In the present study, pretreatment with baicalein significantly decreased the cisplatin-induced MPO activity, a surrogate marker which is linearly related to infiltration of inflammatory cell in inflamed tissue and the infiltration of inflammatory cells in the kidneys as evidenced by light microscopic examination (H and E staining) of kidney tissue slides. Nuclear factor-kappa B (NF-κB) exists in the cytoplasm as an inactive complex with the inhibitory protein, IκBα. Activation of NF-κB by pro-inflammatory cytokines or oxidative stress involves IκBα phosphorylation by IκB kinase (IKK) and subsequently ubiquitination, allowing NF-κB to translocate to the nucleus, where NF-κB triggers transcriptional activation of the genes related inflammation. Activation NF-κB signaling also plays a key role in mediating inflammation through induction of pro-inflammatory cytokines and other downstream proteins including iNOS and COX-2 [1]. It has been reported that the production of ROS and the activation of JNK and p38 MAPK signaling in response to cisplatin insult induces the renal expression of TNF-α, which further triggers the activation of NF-κB [6, 25]. Indeed, as reported in our previous study, NF-κB activation plays an important role in cisplatin-induced renal toxicity [1]. In the present study, pretreatment with baicalein markedly suppressed the cisplatin-induced pro-inflammatory cytokines release such as TNF-α and IL-6 and attenuated the NF-κB activation through inhibition of IKK phosphorylation and IκBα degradation and subsequently inhibited the cisplatin-induced renal inflammation.

MAPKs are a family of structurally-related serine/threonine kinase enzymes. The extracellular-receptor kinases (ERK1/2), the c-Jun N-terminal kinases (JNK) and the p38 MAPKinases constitute the MAPK family. A growing body of evidence suggests the MAPK pathway is a critical axis in regulating cisplatin-induced renal cell death and inflammation [50]. Depending upon the stimulus, the cell type and the duration of exposure, a variety of biological responses are associated with MAPKs activation [51]. There is considerable evidence showing that activation of JNK and p38 MAPK through production of ROS and TNFα in renal cells during cisplatin exposure play important roles in the regulation of apoptosis and inflammatory process [52, 53]. Moreover, literature also suggests that cisplatin exposure activates ERK1/2 to induce renal cell apoptosis through up-regulation of Bax and p53 and mitochondrial cytochrome c release to activate caspase-3 [51]. In this contest, we observed a significantly increased amount of phosphorylated ERK1/2, JNK and p38 proteins in kidneys of cisplatin treated mice after 72 h of cisplatin exposure. Pretreatment with baicalein blocked the activation of ERK, JNK and p38, thereby providing a potential mechanism for the beneficial effect of baicalein. These findings corroborate those of earlier studies in which p38 [52], JNK [53] and ERK1/2 [54], phosphorylation increased after cisplatin administration and inhibition of these reduced tissue damage and improved renal function in vivo. Coming to the pharmacokinetic profiles of baicalein, studies from different research group reported that baicalein is an active endosomatic constituent [13]. After oral administration of baicalein, it rapidly changes into glucuronide/sulfate conjugates and represents the major metabolites in the blood stream, whereas baicalein itself was negligible. It is reported that oral bioavailability of baicalein was 36.1 ± 4.4% in normal rats. The C_max_ of baicalin (a major baicalein conjugate) (5.0 ± 0.7 nmol/mL) was reached at 0.5 h with a long elimination half-life (t_1/2_) of 11.8 h [55].

In conclusion, the results of the present study indicate that baicalein may represent a novel promising approach for the prevention of cisplatin-induced renal impairment. The protective effects of baicalein seem to be due to down regulation of oxidative stress, apoptosis and inflammation via up regulation of Nrf2/HO-1 proteins and inhibition of MAPK activation and NF-κB signaling pathways.
